# Centrifugal microfluidics for rapid target analyte quantification in airborne bioaerosols

**DOI:** 10.1039/d6lc00061d

**Published:** 2026-06-05

**Authors:** Soongwon Cho, Michael Brothers, Ziyu Chen, Samet Şahin, Yirui Xiong, Nicole Schaeublin, Doug Adkins, Charles Call, Anthony Banks, Steve S. Kim, John A. Rogers

**Affiliations:** a Querrey Simpson Institute for Bioelectronics, Northwestern University Evanston IL 60208 USA jrogers@northwestern.edu; b Center for Bio-Integrated Electronics, Northwestern University Evanston IL 60208 USA; c Department of Chemical Engineering, College of Engineering, Kyung Hee University Yongin 17104 Republic of Korea; d Air Force Research Laboratory 711th Human Performance Wing, Wright Patterson Air Force Base Dayton OH 45432 USA steve.kim.13@us.af.mil; e BlueHalo 4401 Dayton-Xenia Road Dayton OH 45432 USA; f Department of Material Science and Engineering, Northwestern University Evanston IL 60208 USA; g College of Engineering and Computer Science, The Australian National University Canberra Australian Capital Australia; h School of Engineering, Lancaster University Lancaster LA1 4YW UK; i Defiant Technologies 6814 Academy Pkwy W NE Albuquerque NM 87109 USA; j Bioflyte 10510 Research Rd SE #100 Albuquerque NM 87123 USA; k Wearifi 191 Waukegan RD Ste 365 Northfield IL 60093 USA; l Department of Biomedical Engineering, Northwestern University Evanston IL 60208 USA; m Department of Neurological Surgery, Northwestern University Evanston IL 60208 USA

## Abstract

Bioaerosols comprise an array of airborne particles that can be benign, contain irritants (*i.e.*, allergens), or disseminate pathogens. Current fieldable devices are incapable of discriminating between benign bioaerosols and pathogenic bioaerosols. Thus, rapid on-site detection and identification at the point of exposure of the components comprising bioaerosols is critical to assess health risks; elimination of both false positives and false negatives is essential to ensure that the correct countermeasures are deployed. The sandwich enzyme-linked immunosorbent assay (sandwich ELISA) remains a gold standard technique for accurate, sensitive detection of proteins, bacteria, and viruses. ELISAs require recognition of the target by two biorecognition elements (BREs) and thus effectively eliminate false positives. The sandwich ELISA, however, suffers from laborious manual processes, which severely impacts its utility in point-of-care (POC). Centrifugal microfluidics offers an automated solution to execute multi-step ELISAs. Here, we introduce a centrifugal microfluidic device that operates based on passive capillary and siphon valving networks to detect human serum albumin (HSA) concentrations in collected bioaerosol samples. We investigate key aspects for minimizing the assay operation time by investigating siphon valve mechanisms, co-incubation time for HSA with a capture antibody and detection antibody conjugated with streptavidin-horse radish peroxidase (strep-HRP), and incubation time for tetramethylbenzidine with strep-HRP for electrochemical amperometric detection. The fully integrated device quantifies HSA concentrations in 15 minutes based on a small sample volume of 12.5 μL. Using the device, we successfully quantify HSA concentrations in bioaerosol samples and obtain results similar to standard 96 well plate-based ELISA.

## Abstract 2

The PDMS-based lab-on-a-CD ELISA platform enables rapid on-site detection of airborne bioaerosols with minimal sample volume.

## Graphical abstract

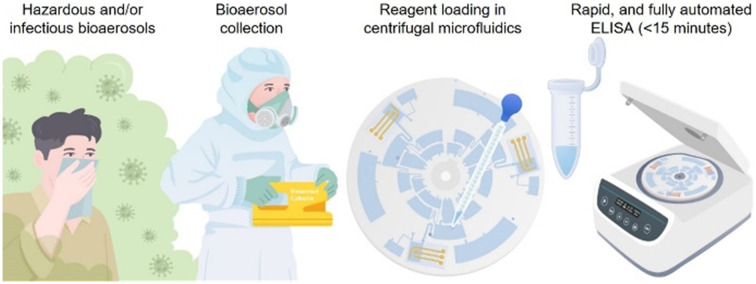

## Introduction

In recent decades, medical and clinical diagnostics to detect pathogens and toxins of interest have become more available, cheaper, and easier to use, allowing their deployment for quarantining of individuals, such as during the COVID-19 pandemic which accounted for 2.6 million deaths in 2020.^1^ However, these methods require sampling of an individual's biofluids post-infection and do not inform on the viral shedding of the individual, and thus how contagious an individual is at a moment in time; this resulted in sub-optimal quarantining, as proximity to an infected individual is used as a proxy for likelihood of viral spread, and not actual exposure to the infectious agent *via* small airborne particles (bioaerosols). Therefore, improved methods are needed to rapidly collect and identify bioaerosols containing compounds of interest.

Bioaerosols are micrometer to nanometer sized airborne particles consisting of a heterogeneous mixture of water, minerals, and biological matter. Bioaerosols can contain one or more of the following: living or dead organisms including bacteria, fungi, and viruses, proteins including biotoxins and pollen, and biomolecules including endotoxins and mycotoxins.^2^ Their typical concentrations are often observed in ranges of 10^2^–10^6^ colony forming units per m^3^ for bacteria and fungi^3^ and 1–2000 grains per m^3^ for pollen.^4^

Various natural and artificial sources produce bioaerosols such as microbial degradation, living animals or humans, and human activities or processes.^2,5^ Inhalation of such toxic or infectious bioaerosols can negatively impact public health and cause allergies, respiratory disorders, inflammation, infection, and acute health issues.^2,5–9^ However, the threat posed by bioaerosols depends on the components contained within the bioaerosol. Therefore, rapid identification of the bioaerosol components, particularly those that are pathogenic and/or toxic, is required to assess the health risk and inform on the correct mitigation/countermeasures to be deployed. More specifically, rapid on-site detection of toxic and hazardous target analytes within bioaerosols is critical for preventing infectious respiratory diseases, biodefence, and monitoring the exposure risk of workers in occupational environments.^10^

Unfortunately, reagentless, optical methods, such as Raman spectroscopy,^11^ to date do not have the requisite specificity to identify toxic agents present in bioaerosols. Therefore, current methods rely on collecting bioaerosols before performing a specific diagnostic test. Aerosol collection often uses a pump to accelerate the bioaerosol particles at a target of interest, either physically entrapping the particles or creating an aerodynamic trap that deposits the larger aerosol particles into a liquid or onto a wall. Collection of bioaerosols^12^ can be performed either onto a filter, a solid surface (impaction), or into a liquid (impingement), allowing for downstream analysis after transfer using mass spectrometry (MALDI^13,14^ or ESI^15^), molecular diagnostics, or immunoassays.

Pathogens, including viruses, bacteria, and fungi, are most accurately and sensitively detected using methods leveraging the polymerase chain reaction (PCR).^16^ Next-generation detectors can reduce the complexity of devices needed to execute the diagnostic assay using isothermal methods such as loop-mediated isothermal amplification (LAMP) and can use multiple colorimetric^17^ or fluorometric^18^ probes to enable multiplexing; however, PCR based methods require DNA/RNA, and are thus limited to organisms. As a result, proteins, including pollen, and small molecules, including endotoxins, must be detected using immunoassays.

Multiple transportable platforms have been developed that enable immunoassays at the point-of-care. Immunoassays can either use a single biorecognition element (BRE) to bind and precipitate the target analyte or two orthogonal BREs to create a sandwich; either platform can then have the binding event correlated with an optical or electrochemical signal. The classical protein immunoassay requires multiple steps to create a signal, consisting of 1) analyte capture and immobilization by a primary, immobilized BRE, 2) washing, 3) analyte binding by a secondary BRE, 4) washing, and 5) signal readout/generation. In some cases, this can be done using a paper matrix, such as for lateral flow assays (LFAs) for proteins including human chorionic gonadotropin (hCG) in pregnancy tests, where the readout can then be reported as a change in absorbance (colorimetric or fluorometric) or reflectance (fluorometric). However, matrix effects are known to cause variability in immunoassay readouts, and thus LFAs can be prone to false positives and negatives^19^ when the fluid sample changes in composition.

The preferred immunosensor modality is one in which the binding event triggers the sensor change, creating a “reagentless system” and thus enabling continuous use of the sensor. Reagentless optical platforms leverage changes in material surface properties that cause shifts in resonance as a function of mass, such as surface plasmon resonance (SPR) and surface-enhanced Raman spectroscopy (SERS). Reagentless electrochemical based assays include field-effect transistors (FETs), electrochemical aptamer-based sensors (E-AB), and impedimetric sensors that use electrochemical impedance spectroscopy (EIS). However, none of the platforms listed work optimally with antibodies due to the size (large) and rigidity of the capture BRE. Additionally, since these single BRE platforms rely on the affinity and selectivity of a single BRE, identification of alternative BREs that have sufficient affinity and selectivity, including DNA aptamers and peptides, remains a substantial challenge. Table 1 summarizes the pros and cons of current rapid methodologies for detecting specific target analytes in bioaerosols.^20^ Therefore, the two-BRE system remains the gold-standard, especially when eliminating false positives and negatives, such as for bioaerosol monitoring and biosurveillance, is essential.

**Table 1:** Pros and cons of current rapid methodologies to detect proteins

Detection methods	Pros	Cons
Field effect transistor (FET) immunosensor	Reagentless	Highly sensitive to changes in buffer, salinity, and interferents
	Rapid response and high sensitivity	Binding events must be close to the surface; typically require a synthetic biorecognition element (BRE) (peptide, aptamer) with significant charge density
	Small form factor	A single BRE limits selectivity
Electrochemical aptamer-based sensor (E-AB) immunosensor	Reagent-less	Requires a structure-switching oligonucleotide aptamer modified with a redox mediator (*i.e.*, methylene blue). Few E-AB sensors for proteins exist (NPY,^32^ thrombin^33^)
	Small form factor	A single BRE limits selectivity and limit of detection
Electrochemical impedance spectroscopy (EIS) immunosensor	Rapid response	Complicated electronics and analysis
	Zero or one reagent needed	Limit of detection and selectivity determined by a single BRE
	Small form factor	Works best when the capture BRE is small (synthetic) and the analyte is large
Optical-based immunosensor (surface plasmon resonance (SPR), surface enhanced Raman spectroscopy (SERS))	Can identify binding as well as quantify underlying binding properties (thermodynamics)	Only use a single BRE, thus may lack selectivity
	Flow cells and chips can be easily replaced in the field	Fieldable units have reduced/insufficient resolution and sensitivity
	Rapid response to analytes	Buffer mismatch can create optical artefacts, especially for real-world samples
Lateral flow assay	Rapid response (<15 minutes)	Relatively low sensitivity (mg mL^−1^) compared to ELISA (reagent delivery)
	Easy to use form factor	Device-to-device reproducibility is difficult
	Improved specificity using 2 BREs (ELISA)	Quantification struggles when changes exist in the buffer/matrix
ELISA	Highly sensitive (ng mL^−1^)	Multiple steps require manual processing/time
	Highly selective using 2 BREs	Assay output takes hours
	Minimal background interference	Some outputs require expensive readers

Among two-BRE sandwich assays, enzyme-linked immunosorbent assay (ELISA), where an enzyme is directly or indirectly attached to the detection BRE, is preferred over direct optical detection because of the amplification of signals that results from the presence of an enzyme reporter. The most common reagent/enzyme combination for ELISA is the use of the enzyme horseradish peroxidase (HRP) coupled to 3,3′,5,5′-tetramethylbenzidine (TMB) in the presence of hydrogen peroxide (H_2_O_2_). In short, the HRP couples the reduction of H_2_O_2_ to the oxidation of TMB, creating a colorimetric change (clear to blue) that correlates to both the concentration of HRP present (and thus detection antibodies present) and the reaction time. Notably, small concentrations of HRP can result in large colorimetric changes due to the amplification of signals driven by the enzymatic reaction.

However, ELISA also suffers from multiple assay steps with manual operations, laborious washing processes, a long assay time, high cost, and bulky readout equipment.^21^ Therefore, developing an automated ELISA with rapid readout capability is required for point-of-care monitoring of a specific target analyte within bioaerosols.^22,23^ Centrifugal microfluidics, operating based on centrifugal force, offer solutions for automating multiple sequences of ELISA including reagent incubation, washing, mixing, and sequential addition of reagents.^22,24,25^ Enhanced mixing processes enable rapid detection of target analytes based on minimal sample volume (<15 μL) within 15 minutes.^26,27^Table 2 summarizes recent advances in centrifugal microfluidic platforms for immunoassay and demonstrates that our platform is comparable to the current state-of-the-art platforms in terms of its detection time, reagent volume, fully passive mode of operation, and sensitivity, while noting that direct comparison of limits of detection depends on the target biomarker, antibody pair, and assay chemistry.

**Table 2:** Comparison with the state-of-the-art centrifugal microfluidics literature studies

Parameters	Lab-on-a-disc for fully integrated ELISA	Flow-enhanced electrochemical immunosensors	Lab in a bento box	This paper
Published	2012	2013	2020	2026
Sample matrix	Whole blood	DPBS	DPBS	Bioaerosol
Fluidic control	Active	Active	Passive	Passive
	(Wax valve with laser)	(Wax valve with laser)	(Centrifugal hydrokinetics)	(Capillary/siphon)
Throughput	3	3	2	3
Target biomarker	C-reactive protein	C-reactive protein	Mouse IgG	Human serum albumin
	Cardiac troponin I			
	N-terminal			
	Pro-B type natriuretic peptide			
Detection time	20 minutes	20 minutes	12 minutes	15 minutes
Sample volume	66 μL	75 μL	30 μL	12.5 μL
Detection antibody	66 μL	75 μL		12.5 μL
Washing volume	116 μL	200 μL	150 μL	60 μL
TMB volume	100 μL	110 μL	40 μL	30 μL
Limit of detection	0.27/0.46/0.34 ng mL^−1^	4.9 pg mL^−1^	0.76 ng mL^−1^	4.2 ng mL^−1^
Detection	Optical	Electrochemical	Optical	Electrochemical
Reference	24	22	26	This work

Here, we introduce a centrifugal microfluidic platform based on passive capillary and siphon valving networks for an automated ELISA after manual sample loading. Human serum albumin (HSA) was used as a model target analyte, as its size (66.5 kDa) is comparable to pollen,^28^ protein biotoxins,^29^ and other protein targets of interest. First, we demonstrate the capabilities of performing fluidic operations entirely based on centrifugal force without active actuation. We then investigate key components of the device for minimizing the assay time including siphon valves, co-incubation time for HSA with capture and detection antibodies, and TMB incubation time with HRP enzymes. Through these optimizations, the device successfully detects HSA concentrations in collected bioaerosol samples (12.5 μL) within 15 minutes with sensitivity comparable to that of plate ELISA.

## Results

### Device architecture, use cases, and bioaerosol collector

**Fig. 1:**
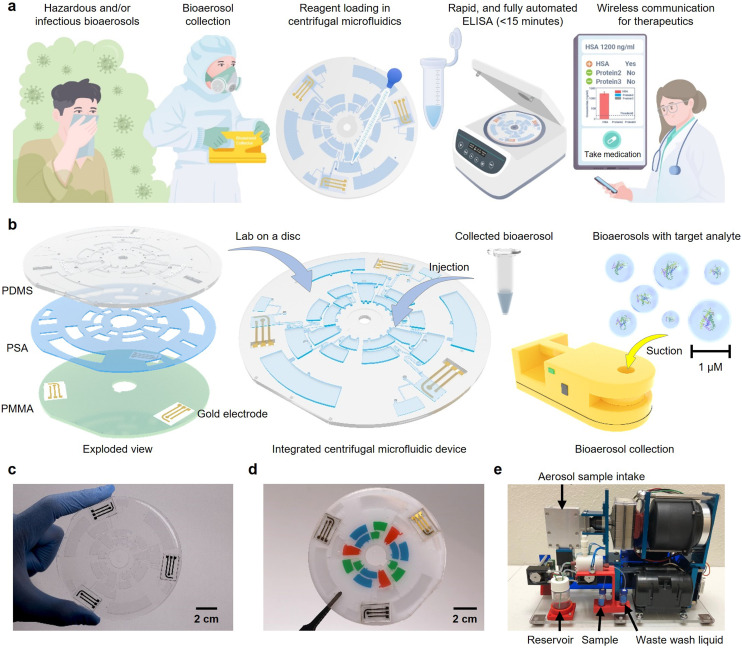
Schematic illustrations and images of the centrifugal microfluidic platform for rapid on-site bioaerosol detection. a) Cartoon illustrations of collection of hazardous bioaerosols, injection into a centrifugal microfluidic device, an automated enzyme-linked immunoassay (ELISA) on the device, and rapid readout (<15 minutes) of protein concentrations. b) Exploded view schematic illustration of a device for ELISA, its integration for ELISA, and bioaerosol collection. c) Photograph of a device with a transparent polymethyl methacrylate (PMMA) substrate for ELISA, scale bar 2 cm. d) Photograph of a device with a white PMMA substrate with food dyes for visualization of the microfluidic chambers, scale bar 2 cm. e) Photograph of the bioaerosol collector provided by BioFlyte/Defiant Technologies. The collector is a separate upstream module from the centrifugal microfluidic device. The collector contains a single-stage axial impactor adjacent to the aerosol sample intake, allowing collection into a sample cup. Buffer from the reservoir re-suspends collected aerosols into the sample outlet or the waste wash liquid collection vial.

Fig. 1a depicts the use of a centrifugal microfluidic platform for monitoring toxic and hazardous bioaerosols in an environment. The process involves collecting bioaerosols using an external impactor, resuspending the collected material in liquid buffer, manually injecting the liquid sample into the centrifugal microfluidic platform, and quantifying protein levels through an automated ELISA after manual sample loading, as the basis for guiding responsive action. The readout can be obtained within 15 minutes using portable equipment, allowing timely intervention.

The device consists of four components (Fig. 1b, left): a slab of polydimethylsiloxane (PDMS) with relief structures that define channels and chambers (microfluidic layer), a synthetic pressure sensitive adhesive (PSA) with CO_2_ laser-cut microfluidic chambers, a bottom substrate of polymethyl methacrylate (PMMA), and gold electrodes on a glass substrate. These layers bond together to create a series of reservoirs that can be modified to enable fluidic movement, and thus operation of an ELISA (Fig. 1b, middle). The input for this lab-on-a-CD consists of bioaerosols collected using a sampler, such as the handheld Biocapture 650 (Fig. 1b, right), that captures airborne particles using a rotating impactor into a water-based collection fluid. Fig. 1c and d show the assembled device and with food dyes loaded into each chamber for visualization, respectively. The image of the bioaerosol collector is shown in Fig. 1e, with the ability to concentrate bioaerosols into small volumes that can be processed by the lab-on-a-CD ELISA.

Fig. 1e shows the bioaerosol sample collection instrumentation, which operates as a separate upstream module from the centrifugal microfluidic device and concentrates bioaerosols into small liquid volumes for subsequent centrifugal microfluidic ELISA analysis. The collector consists of an aerosol sample intake inlet adjacent to a single-stage axial impactor milled out of stainless steel plates. The impactor pulls air through the aerosol sample intake and through the spotting nozzle into a sample cup, where an aerodynamic trap causes deposition of bioaerosols in the sample cup. This cup connects to two peristaltic pumps and a series of three-way valves to enable fluid flow in and out to 1) resolubilize the collected bioaerosol in the buffer from the reservoir, 2) move the fluid from the cup into the sample vial, 3) rinse the cup with reservoir buffer and 4) move the waste wash into the waste wash reservoir. This process allows for automated collection of bioaerosols into a buffered solution compatible with bioassays as well as regeneration of the collection system.

### Working principles of the sandwich ELISA assay and the corresponding microfluidic device

**Fig. 2:**
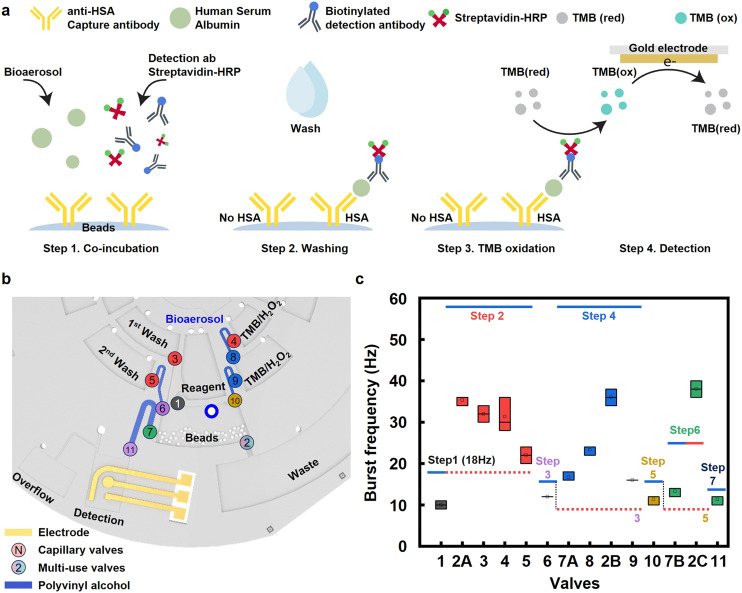
Principles and fluidic logic of centrifugal microfluidic ELISA. a) Schematic illustration of the principles of the sandwich ELISA for HSA detection involving step 1: co-incubation of reagents, step 2: washing, step 3: TMB reaction with strep-HRP, and step 4: electrochemical detection. b) Schematic illustration of the centrifugal microfluidic design with detailed information on reagent loading, capillary valve numbering, siphon valve numbering, polystyrene beads, and gold electrodes for electrochemical detection. c) Burst frequencies of the numbered capillary and siphon valves and detailed spin protocol of the centrifugal microfluidic operation for an automated ELISA. *n* = 3.

Fig. 2a shows the working principles of the bead-based sandwich ELISA used for HSA detection.

In step 1, the capture antibody conjugated polystyrene (PS) beads co-incubate with HSA, biotinylated detection antibody, and strep-HRP. During co-incubation, HSA binds to the capture antibodies on the PS beads, while the biotinylated detection antibody binds to bead captured HSA to form the sandwich immunocomplex. Strep-HRP then associates with the biotinylated detection antibody and serves as the enzymatic label for subsequent TMB oxidation and electrochemical detection. The PS beads serve as the solid phase support for target capture, analogous to the antibody coated surface in a conventional well plate ELISA, while enabling efficient mixing during centrifugal operation. In step 2, the washing step removes any unbound HSA, biotinylated detection antibody, and strep-HRP from the chamber. In step 3, bead bound strep-HRP oxidizes TMB. In step 4, gold electrodes detect the oxidized TMB species by producing amperometric signals proportional to HSA concentrations.

The corresponding device configuration, detailed valve numbering, burst frequencies, and spin protocol are shown in Fig. 2b and c and Table 3. As shown in Fig. 2b, the device consists of a series of chambers for loading and processing assay reagents. Specifically, the reagent chamber contains the bioaerosol sample, biotinylated detection antibody, and strep-HRP for the protein binding reaction. The reaction chamber at the center of the device contains PS beads, where the ELISA reactions occur. The selected bead size balances the capture surface area, mixing efficiency, and reliable retention within the reaction chamber during centrifugal operation. Smaller beads can provide a larger effective surface area and accelerate binding kinetics by minimizing the distance needed for analyte/reagent diffusion.^30^ Bead transport and retention during centrifugal washing also depend, however, on particle size. Stokes-type sedimentation in centrifugal microfluidic systems scales with bead radius squared. As a result, further reductions in bead size may require increased rotational frequencies or spin times for stable retention,^31^ with additional risks of clogging the capillary valves during operation. The number of the valves corresponds to their order of operation during the automated assay sequence. Careful design of the geometry and burst frequency of each valve allows selective actuation of the required valves at specific rotational frequencies during the spin protocol, while the remaining valves remain closed until their designated operation steps, as summarized in Fig. 2c and Table 3.

**Table 3:** Spin protocol for centrifugal microfluidics after loading the collected bioaerosol sample and assay reagents into the device

Step	Description	Frequency (Hz)	Time (s)	Activated valves
1	Conjugation	14	20	1
Mixing		±5	180	
2	First washing	60	90	2, 3, 4, 5
3	Second washing	2	60	
		14	5	6
		10	60	6
Mixing		±5	30	
4		60	90	2, 8, 9
5	TMB substrate reaction	2	60	
		14	5	10
		10	60	10
Mixing		±5	180	
6	TMB detection	25	5	7
		2	60	
7		14	20	11

### Step-by-step sequences of fluidic operation for ELISA through high-speed video imaging

**Fig. 3:**
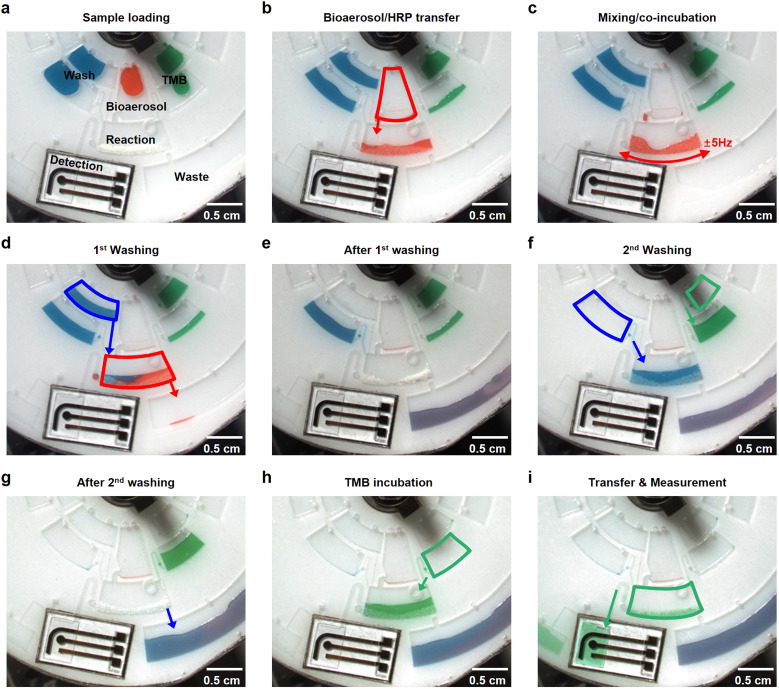
Step by step images from the high speed video for fluidic sequencing characterization. a–i) High-speed stroboscopic images of a device to demonstrate the microfluidic operations. a) Loading of bioaerosol samples with human serum albumin (HSA) (red), washing buffer (blue), tetramethylbenzidine (TMB)/hydrogen peroxide (H_2_O_2_) substrates (green), and polystyrene (PS) beads (white) in the device, scale bar 0.5 cm. b) Transfer of the bioaerosol sample from the loading chamber to the reaction chamber containing PS beads with the conjugated anti-HSA capture antibody, scale bar 0.5 cm. c) Mixing at ±5 Hz and co-incubation of reaction mixtures, scale bar 0.5 cm. d) Transfer of the bioaerosol sample from the reaction chamber to the waste chamber and 1st washing of the chamber by laminar flow, scale bar 0.5 cm. e) Image of the device after 1st washing, scale bar 0.5 cm. f) Transfer of the wash buffer for 2nd washing and transfer of TMB/H_2_O_2_ from the upper siphon to the lower siphon chamber, scale bar 0.5 cm. g) Image of the device after 2nd washing, scale bar 0.5 cm. h) Transfer of TMB/H_2_O_2_ redox probe solution to the reaction chamber, scale bar 0.5 cm. i) Transfer of the reacted TMB/H_2_O_2_ solution to the detection chamber for chronoamperometric sensing, scale bar 0.5 cm.

A series of high-speed camera images (E2E-S05S12-WC-C1, Omron) show the operating principles of the fluidic operations for the device (Fig. 3a–i). In this study, liquid assay reagents were loaded into the CD shortly before operation rather than stored long-term on-disc. Capture antibody-conjugated PS particles were stored in solution at 4 °C and remained stable for more than 1 month. First, the bioaerosol sample with HSA as a model protein target and assay reagents pass into each of the chambers using a pipette before spinning the disc (Fig. 3a). Spinning the disc at 14 Hz for 20 seconds transports the HSA-containing bioaerosol sample (red) mixed with strep-HRP into the reaction chamber which contains 5 mg of anti-HSA antibody conjugated PS beads (Fig. 3b). Shaking the disc at ±5 Hz with 100 Hz per second acceleration for 3 minutes facilitates mixing of the bioaerosol sample with the PS beads and the binding reaction of HSA to the capture antibody (Fig. 3c). Appropriate rotational speed, zero dwell time, and fast rotational acceleration or deceleration are critical for achieving good mixing within the chamber. After conjugation, two separate washing steps ensure the complete washing of the PS beads in the reaction chamber. The first washing step involves rotating the disc at 60 Hz for 90 seconds (Fig. 3d). This process breaks the capillary valve for the reservoir containing the washing buffer. The washing buffer then runs through the reaction chamber to the waste chamber with the reaction mixture (Fig. 3e). The second washing step involves priming the washing buffer solution through the burst siphon valve with local polyvinyl alcohol (PVA) treatment at 2 Hz for 60 seconds (Fig. 3e). Subsequent rotation at 14 Hz for 5 seconds breaks the capillary valve between the siphon valve and the reaction chamber. Next, rotation at 10 Hz for 60 seconds completely transfers the washing buffer to the reaction chamber (Fig. 3f). Mixing the beads with washing buffer involves shaking the disc at ±5 Hz with 100 Hz per second acceleration for 30 seconds. Spinning at 60 Hz for 90 seconds moves the washing buffer to the waste chamber (Fig. 3g). Fig. 3h shows the transfer of the TMB/H_2_O_2_ substrate solution (green) to the reaction chamber. The spin protocol for the TMB transfer step is identical to the second washing step, which involves priming the TMB solution through the siphon valve at 2 Hz for 60 seconds, initiating the transfer at 14 Hz for 5 seconds, and completing the transfer process at 10 Hz for 60 seconds. Mixing at ±5 Hz ensures the reaction between TMB/H_2_O_2_ and strep-HRP enzymes on PS beads within 3 minutes.

The oxidized TMB/H_2_O_2_ substrate solution passes to the chamber for amperometric detection *via* a series of spin controls (Fig. 3i). The siphon valve from the reaction chamber to the detection chamber is activated by spinning the disc at 25 Hz for 5 seconds. Spinning at 2 Hz for 60 seconds primes the siphon valve. Rotation at 14 Hz for 20 seconds transfers the sample to the detection chamber. A demonstration of the automated fluidic process appears in Video S1.

### Development of centrifugal microfluidic ELISA for rapid protein quantification

The total operation time for the bead-based centrifugal microfluidic ELISA is 15 minutes from the start of the assay to the detection after loading the collected bioaerosol sample and assay reagents into the device (Table 4). The total bioaerosol sampling to result workflow additionally includes upstream bioaerosol collection and manual reagent loading into the device, which require 10 minutes and 2 minutes, respectively. Thus, the complete workflow requires approximately 27 minutes under the conditions used in this study. Table 4 compares the performance parameters of the bead-based centrifugal ELISA developed in this work with those of the conventional planar 96 well-plate ELISA format based on the DuoSet ELISA kit protocol (DY1455). For the bead-based centrifugal microfluidic assay, fluidic operations, protein binding events, washing processes and TMB oxidation reactions require 4, 3, 5 and 3 minutes, respectively.

**Table 4:** Comparison with traditional ELISA

Parameters		Conventional planar 96 well plate HSA ELISA	This work
Assay format	Format	96 well-plate planar sandwich ELISA	Bead-based sandwich ELISA
	Capture support	Antibody coated well-plate surface	Antibody conjugated PS beads
	Operation	Manual pipetting	Automated centrifugal microfluidics
	Detection method	Optical	Electrochemical
Assay preparation	① Bioaerosol collector	10 minutes	10 minutes
	② Sample loading	2 minutes	2 minutes
Assay time	③ Fluidics/operation	10 minutes	4 minutes
	④ Protein binding	2 hours	3 minutes
	⑤ Washing	30 minutes	5 minutes
	⑥ TMB oxidation	20 minutes	3 minutes
	Total	3 hours 12 minutes	27 minutes
Reagent volume	Sample volume	100 μL	12.5 μL
Limit of detection		2.5 ng mL^−1^	4.2 ng mL^−1^

**Fig. 4:**
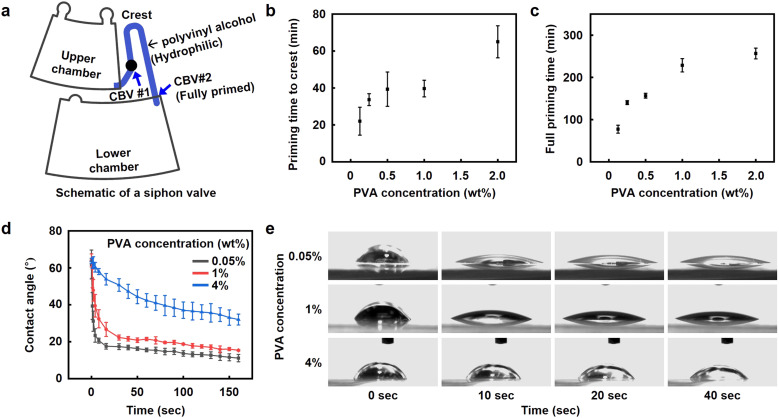
Centrifugal microfluidic siphon valve characterization. a) Schematic illustration of a siphon valve defining terminologies. b) The effect of drop-casted polyvinyl alcohol (PVA) concentrations on priming time to the crest location. *n* = 3. c) The effect of drop-casted PVA concentrations on time for fully priming a siphon valve. *n* = 3. d and e) Change of contact angle over time for a 2 μL water droplet drop-casted on a PVA-coated polydimethylsiloxane (PDMS) surface with varying weight percentages. Thin films of PVA are created by drop-casting 20 μL PVA solutions of 0.05, 1, and 4 wt% on plasma treated PDMS surfaces (12 mm by 20 mm). *n* = 3.

Optimizing the siphon valve is crucial for reducing the time required for fluid operation. The time for fluids to travel through the hydrophilic siphon microchannel can vary from 1 minute to more than 4 minutes depending on the characteristics of the PVA coating. Measurements of this priming time involve observations of the times required for the fluid to reach the crest and the full length of the siphon valve after bursting capillary valve 1 (Fig. 4a). Increasing the concentration of PVA on the microchannel decreases the rate of the capillary hydrophilic wicking process due to the reduced rate of PVA dissolution at the moving liquid front (Fig. 4b and c). Fig. 4d and e display the dynamic change in the contact angle of a water droplet on a PDMS surface coated with a thin film of PVA. The contact angle decreases gradually over time due to the slow dissolution of PVA in the water at high PVA concentration which is consistent with observations of the siphon valve priming time. We evaluated the effect of temperature on siphon priming time because PVA dissolution and liquid viscosity can depend on ambient temperature. Siphon priming time decreased with increasing temperature, from approximately 46 s at 0 °C to 29 s at 20 °C and 19 s at 40 °C (Fig. S5). Although temperature affected valve timing, the siphon primed within less than 1 min across the tested range, indicating that the current spin protocol provides sufficient timing margin for valve operation.

**Fig. 5:**
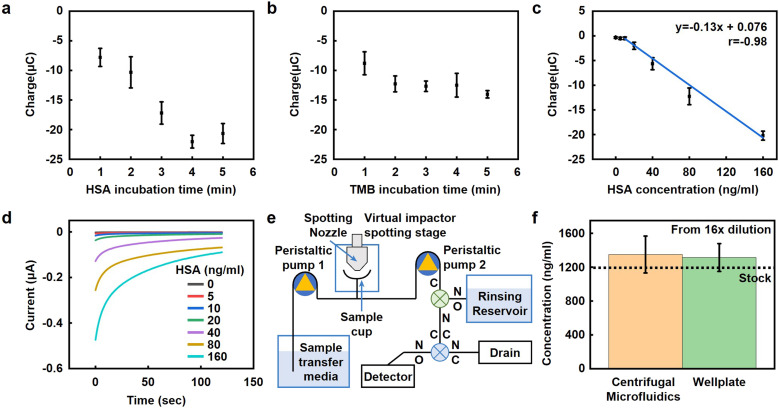
Development and characterization of centrifugal microfluidic ELISA. a) The effect of co-incubation time (step 1) on the chronoamperometric charge at 2 minutes for 80 ng mL^−1^ HSA. *n* = 3. b) The effect of TMB incubation time (step 3) on the chronoamperometric charge at 2 minutes for 80 ng mL^−1^ HSA. *n* = 3. c) Standard calibration curve of sandwich ELISA for detection of HSA in a reagent diluent. *n* = 3. d) Chronoamperometric ELISA response to HSA concentrations of 0, 5, 10, 20, 40, 80, and 160 ng mL^−1^. *n* = 1. e) Schematic of how bioaerosol is 1) collected onto the sample cup surface, 2) re-solubilized in buffer from the liquid reservoir, and 3) moved to the detector using vacuum pumps, peristaltic pumps, and valving. f) Detection of HSA concentrations in collected bioaerosol samples with plate and centrifugal microfluidic ELISAs. *n* = 3.

Detailed investigations reveal that an incubation time of 3 minutes for both step 1 (HSA co-incubation) and step 3 (TMB oxidation) is sufficient to generate a detectable signal for HSA at a concentration of 80 ng mL^−1^ (Fig. 5a and b). The saturation behavior of the measured charge signal defined the criterion for selecting each incubation time. Three minutes correspond to the time beyond which the rate of change in charge drops below a threshold value. After this time, continued incubation produces negligible signal gain relative to measurement variability. This response follows from the large surface area of the PS beads, together with an effective centrifugal mixing process based on optimized spin protocols. Owing to the digitally controlled spin protocol, the actual timing error in practice is on the order of 1 second or less. Even under a conservative assumption of a ±5 second deviation, the resulting variation in the measured signal remains within ±2.5% for protein binding and ±0.4% for TMB oxidation, confirming that the assay is insensitive to minor timing fluctuations. A standard calibration curve based on 3 minute incubation time reveals a limit of detection (LOD) of 4.2 ng mL^−1^ and a linear range of 10 to 160 ng mL^−1^ with a correlation coefficient of 0.99 (Fig. 5c). This value is comparable to the lower assay range of the conventional HSA Duoset ELISA, 2.5 ng mL^−1^, while using a substantially shorter assay time. Further improvements in LOD may be possible by optimizing bead loading, incubation conditions, and electrochemical signal amplification. The corresponding chronoamperometric responses for the standard calibration curve are shown in Fig. 5d. This assay can be conducted on volumes as small as 12.5 μL, compatible with short bioaerosol collection times. Fig. 5e illustrates the use of peristaltic pumps and valves to move the resolubilization buffer into and out of the sample cup containing the bioaerosol. Notably, valve operation allows the buffer from the liquid reservoir or a separate rinsing reservoir to 1) resuspend the protein bioaerosol for analysis, 2) agitate by vibration and/or fluid pumping, 3) transfer the collected bioaerosol to the device for analysis and 4) wash the sample cup before subsequent bioaerosol collections. Detailed collection times and volumes during sample rinsing and washing steps are included in Table S1. Fig. 5f demonstrates the ability to estimate HSA concentrations in collected bioaerosol samples, with comparisons to results obtained with standard plate ELISA methods. To enable quantitation within the lab-on-a-CD, collected bioaerosol samples are diluted 16 times before use in both plate and centrifugal microfluidic ELISAs. The estimated HSA concentrations of these diluted bioaerosol samples are 84.3 ng mL^−1^ and 82.1 ng mL^−1^ for centrifugal microfluidics and plate ELISA, respectively.

## Conclusion

Here, we present a platform for rapid quantification of target analytes in captured bioaerosols within an automated centrifugal microfluidic system. First, we show that protein bioaerosols can be collected and re-solubilized into aqueous media using a single-stage axial impactor followed by rinsing and agitation of a dry sample cup. Notably, the collected protein maintains a sufficient tertiary structure to be recognizable by monoclonal antibodies used in ELISAs. We then demonstrate detection of the protein bioaerosol using an automated ELISA that operates *via* a lab-on-a-CD device. The device consists of PDMS, PSA, and PMMA with robust adhesion between layers through plasma treatment. The PDMS microfluidic layer allows for rapid prototyping, precise microstructure formation through soft lithography, and low-cost device fabrication.

Notably, we illustrate that the device can detect the proteins from the bioaerosol by operating a rapid ELISA. The collected bioaerosol sample is then injected into the device for the quantification of HSA within 15 minutes. Optimization of microfluidic siphon valves, co-incubation time of reagents, and TMB reaction time with strep-HRP enzymes enables rapid HSA quantification. A lower concentration of hydrophilic PVA coating on the microchannels facilitates the liquid priming process which leads to faster priming speed for the siphon valve. This allows the entire microfluidic process to be completed within 4 minutes. Enhanced mixing processes within the device ensure successful HSA quantification with 3 minutes of co-incubation time for all assay reagents, and 3 minutes for the TMB/H_2_O_2_ reaction. Application of the system for detecting HSA concentrations in collected bioaerosol samples demonstrates excellent performance, as validated by gold standard well plate-based ELISA. Initial experiments using AuNPs as model particulate interferents show limited effects on HRP-mediated TMB electrochemical detection and indicate that BSA blocking can reduce particle associated non-specific background. Comprehensive testing with diverse real-world environmental matrices remains an important future direction for field deployability. Other biotoxins or infectious agents can be detected by modifying the platform described here, mainly by swapping out the detection and capture antibodies to enable selective detection of the target analyte; the bioaerosol collector may need to be modified to use liquid impingement to preserve the protein tertiary structure, but otherwise the device from bioaerosol collection to analysis should require minimal changes. Thus, we expect that the technologies introduced here offer a platform for rapid collection and detection of toxic and infectious bioaerosols that will enable improved implementation of countermeasures to reduce the spread of infectious agents, including deployment of quarantines and therapeutics to proactively treat individuals exposed to toxic bioaerosols.

## Materials and methods

### Chemicals and apparatus

Propylene glycol monomethyl ether acetate (PGMEA), ethanolamine, PVA, and trichloro(1*H*,1*H*,2*H*,2*H*-perfluorooctyl)silane were purchased from Sigma Aldrich. Protein LoBind Eppendorf tubes (1.5 mL), glass slides (75 mm × 50 mm), and standard biopsy punches (1 mm) were purchased from Thermo Fisher Scientific. 4″ silicon wafers were purchased from University Wafer. Samples of double-sided medical adhesive (9965, 9877) were provided by 3M. 15 pcs leather hole punch set was purchased from Amazon. A human serum albumin Duoset ELISA kit #DY1455, Duoset Ancillary Reagent kit 2 including plate-coating buffer (1× phosphate buffered saline, PBS), 10% bovine serum albumin (BSA) solution, stop solution (2 N H_2_SO_4_), TMB substrate, stabilized H_2_O_2_, clear 96-well microplates, and plate sealers were purchased from R&D systems. Carbon steel dowel pins (0.4 mm) and clear acrylic sheets (1.57 mm) were purchased from McMaster. Iron oxide blank photomasks were purchased from Nanofilm. An ME-9028 iron oxide etchant was purchased from Transene. AZ10XT and AZ400K developers were purchased from IMM (Integrated Micro Materials). SU-8 2005, 2035, 2050, and 2100, and SU-8 developer were purchased from Kayaku. 1-Ethyl-3-(3-dimethylaminopropyl) carbodiimide hydrochloride (EDC) and *n*-hydroxysulfosuccinimide sodium salt (sulfo-NHS) were purchased from G-Biosciences. ACE 2-morpholinoethanesulphonic acid (MES) buffer (50 mM) was purchased from Bioworld. Carboxy PS beads were purchased from Rapp Polymere.

### Preparation of a soft lithographic mold for microfluidic chambers and channels

Soft lithographic methods applied with molds of a patterned photoresist (SU-8) on silicon wafers produced centrifugal microfluidic structures with the desired characteristics including multiple levels of depth, ranging from 30 to 400 μm, for microfluidic chambers, siphon valves, and capillary valves. The latter involved dimensions smaller than 50 μm to allow access to a wide range of capillary bursting frequencies, corresponding to rotational frequencies at which the valves open. Additionally, large areas (4 inch diameter) were needed to allow for proper centrifugal microfluidic operation. Comparisons of methodologies and materials are shown in Table 5.

**Table 5:** Methods for centrifugal microfluidic device fabrication based on PDMS

Substrate material	Microfluidic device	Bonding	Pros	Cons
PMMA	PDMS	Oxygen plasma	Simple fabrication	Weak bonding
PMMA	PDMS	Oxygen plasma	Strong bonding	Chemical treatment
		(3-Aminopropyl)triethoxysilane		Surface functionalization
		Tetraethyl orthosilicate		
PMMA	PDMS	Thin uncured PDMS	Simple fabrication	Weak bonding
PDMS	PDMS	Oxygen plasma	Simple fabrication	Weak bonding
				Too flexible for spinning
Glass	PDMS	Oxygen plasma	Strong bonding	Glass fragility, safety
				Glass machining
Silicon wafer	PDMS	Oxygen plasma	Strong bonding	Cost of fabrication
				Surface properties
PMMA	PDMS	Rubber-based pressure sensitive adhesive	Strong bonding	—
		(This work)	Rapid prototyping	
			Low cost	
			Microstructures (<30 μm) of multiple levels of depth	

Fabrication of the molds began by defining iron oxide photomasks with a pre-coated AZ1518 photoresist (Nanofilm) using an MLA 150 Maskless aligner (Heidelberg Instruments) with exposure conditions of 375 nm wavelength and 55 mJ cm^−2^ dose. Developing with a 1 : 4 mixture of AZ400K developer (IMM integrated micro materials) and deionized water for 60 seconds exposed the iron oxide layer to allow its patterned removal by etching with ME-9028 (Transene) for 30 seconds. Stripping the residual photoresist by immersion in acetone completed the fabrication of the photomask. Three iron oxide masks were created for different microfluidic thicknesses of 30, 100, and 400 μm.

Preparation of the 4 inch wafer (Universal wafer) began by cleaning with isopropyl alcohol and deionized water and baking on a hot plate at 95 °C for 10 minutes. Plasma treatment (PC300, Samco) at 100 watts for 30 minutes facilitated strong adhesion of SU-8 coatings formed by spin-casting onto the hydrophilic silicon wafer surface. A uniform base layer SU-8 2005 with a 10 μm thickness formed by casting and flood exposure promoted strong adhesion of subsequent SU-8 layers created by multi-layer photolithographic processes consisting of spin-coating, edge bead removal, pre-baking, aligned UV exposure, and post-baking steps. Developing the wafer with the SU-8 developer for 1 hour defined the SU-8 microstructures for molding of the PDMS microfluidic layer. A surface coating of perfluorooctyl trichlorosilane by vapor phase deposition rendered the surface hydrophobic and facilitated the removal of PDMS from the surface of the wafer after curing.

### Fabrication of PDMS microfluidic layers for centrifugal microfluidic operation

Casting 6 grams of PDMS (10 : 1 ratio) on a wafer and curing it on a hot plate at 95 °C for 30 minutes produced 0.8 mm thick layers of PDMS. Rendering the PDMS surface hydrophilic by plasma treatment (PC2000, South Bay Technology) at 100 watts for 30 minutes allowed for local coating of PVA within the microchannels. The hydrophilic surface enabled the PVA solution (1 μL, 0.05% w/w) to spread evenly across the microchannels of the siphon valve upon drop-casting and subsequent drying at room temperature for 30 minutes. A set of mechanical hole punches created inlet holes, vent holes, and rotational center holes for each PDMS microfluidic layer.

### Assembly of devices for ELISA

Using PDMS as a device material enables rapid prototyping, precise formation of small microstructures, and low-cost fabrication. Limited use of PDMS in centrifugal microfluidics results from demanding conditions such as elevated pressures during essential fluidic operations. The specific difficulty lies in identifying suitable substrates for capping the PDMS microfluidic layers, where 1) PDMS substrates cannot provide sufficient bonding, 2) glass substrates are prone to cracking and can be difficult to cut into desired shapes, and 3) polymethyl methacrylate (PMMA, McMaster-Carr) substrates require PSAs (9877, 3MTM). In this work, an intermediate PSA layer and corona treatment enabled strong bonding between PDMS and PMMA, ensuring successful microfluidic operations even at high rotational frequencies exceeding 60 Hz.

Designs for PSA and PMMA layers were prepared in AutoCAD software (Autodesk) and cut using a VLS CO_2_ laser (Universal Laser Systems). A CO_2_ laser system patterned microfluidic chambers with ±254 μm resolution. Soft lithographic processes were used for capillary valves with dimensions smaller than 100 μm. A custom alignment JIG with three pogo pins allowed bonding of the PMMA and PSA layers with a ±200 μm alignment margin. A hard rubber brayer roller (Speedball) ensured tight contact between layers. PMMA/PSA layers served as a substrate for bonding to the PDMS microfluidic layer. Subsequent exposure to a corona discharge activated both surfaces for bonding, with the same JIG for alignment. The device was then baked in an oven at 75 °C for 2 hours to recover hydrophobicity. Placement of electrodes on the detection chamber completed the assembly process.

### Lift-off process for preparation of gold electrodes

The lift-off process began by mixing an AZ10XT positive photoresist (IMM; Integrated Micro Materials) with PGMEA at a 7 : 3 ratio. The photoresist cocktail was then spin-coated onto a glass substrate (75 mm × 50 mm) at 3000 rpm for 30 seconds, followed by a prebake step at 110 °C for 2 minutes. Subsequent exposure to UV light (375 nm, MJB4 mask aligner, Suss Microtec) for 20 seconds and development of the exposed substrate with AZ 300 K developer for 120 seconds created the electrode patterns, preparing the substrate for deposition of 10 nm of chromium and 100 nm of gold layers at a rate of 1 angstrom per second on the photoresist-patterned glass slide using an electron beam evaporator (AJA International). After metal deposition, immersing the glass slide in an acetone bath stripped the AZ 10XT photoresist from the glass slide along with the chromium and gold layers deposited on top of it. The resulting pattern consisted of working (2 mm circle), counter, and reference gold electrodes for the electrochemical detection of the oxidized TMB substrate.

### Collection of bioaerosols

A custom, proprietary single-stage axial impactor allowed collection of the protein bioaerosols (co-developed by Defiant Technologies and BioFlyte). Bioaerosols were generated using agitation from an ultrasonic transducer (vibrating orifice nebulizer) placed into a 20% w/v solution of protein (HSA) in a 2.5 m^3^ chamber. This process introduced ∼6 mg m^−3^ of particles into the chamber.

Bioaerosols were collected at 2 LPM through a single-stage axial impaction using a spotting nozzle (15 mils) to focus the bioaerosol into a collection cup. After collection (10 minutes), a Tris buffer (10 mM Tris, pH 7.5, 0.05% Tween-20) was added to the collection cup to dilute and resuspend the collected bioaerosol from the spotting nozzle into 100 μL of buffer. The solution, once in the spotting nozzle, was agitated using a vibrating motor and heated to a moderate temperature (∼37 °C) to improve the solubilization of the protein. For a five-minute collection time, a maximum of ∼20 μg could be collected and solubilized into 100 μL of buffer.

### Conjugation of the anti-HSA antibody to PS beads

EDC and sulfo-NHS coupling chemistry was used to covalently conjugate the anti-HSA antibody to the carboxylated PS beads. The first step involved washing 10 mg of carboxylated PS bead aliquots three times in LoBind Eppendorf tubes with 0.5 mL of 50 mM MES buffer. The reaction of the carboxylated PS beads with 1 mL of 4.8 mM EDC and 4.8 mM sulfo-NHS activated the beads by a semi-stable amine-reactive NHS-ester formation. The EDC/sulfo-NHS coupling reaction was performed by rotating the beads on a RotoFlex Plus tube rotator (Cole-Palmer) at 20 rpm for 30 minutes at room temperature. Washing the activated beads with 0.5 mL of 50 mM MES buffer twice removed any unreacted EDC/sulfo-NHS from the Eppendorf tube. Subsequent overnight incubation of the activated beads at 35 °C conjugated the anti-HSA antibody (25 μL, 40 μg mL^−1^) to the activated beads. Quenching of the unreacted EDC-NHS esters involved 30 minutes of incubation of the antibody-conjugated beads with 1 mL of 0.5 M ethanolamine. Any remaining surfaces of the PS beads were blocked with 4% BSA solution (0.5 mL) at room temperature for 2 hours on a tube rotator. After blocking, the beads were washed with 0.5 mL of washing buffer three times and injected into the devices for ELISA.

## Author contributions

S. C., M. B., Z. C., and S. Ş. contributed equally to this work. Soongwon Cho: experiment design, device fabrication, data acquisition, data analysis, writing – original draft, and writing – review & editing. Michael Brothers: conceptualization, methodology, project administration, investigation, writing – original draft, and writing – review & editing. Ziyu Chen: experiment design, device fabrication, data acquisition, data analysis, and writing – review & editing. Samet Şahin: experiment design, sensor fabrication, data acquisition, data analysis, and writing – review & editing. Yirui Xiong: schematic illustrations and writing – review & editing. Nicole Schaeublin: investigation and data curation. Doug Adkins: experiment design, device fabrication, data acquisition, and data analysis. Charles Call: experiment design, device fabrication, data acquisition, and data analysis. Anthony Banks: supervision and resources. Steve Kim: conceptualization, resources, supervision, funding acquisition, and writing – review & editing. John A. Rogers: experiment design, supervision, writing – original draft, and writing – review & editing.

## Conflicts of interest

The authors declare that they have no conflict of interest.

## Supplementary Material

LC-026-D6LC00061D-s001

LC-026-D6LC00061D-s002

## Data Availability

All data for this article are available in the main paper or its supplementary materials. The raw datasets generated during the studies are available for research purposes from the corresponding author upon request. Supplementary information (SI) is available. See DOI: https://doi.org/10.1039/d6lc00061d.
