# Supplementary material for: Centrifugal microfluidics for rapid target analyte quantification in airborne bioaerosols
Source: Lab Chip. 2026 Jun 5. Online ahead of print. doi: 10.1039/d6lc00061d (PMC13277543; doi:10.1039/d6lc00061d)
Supplement: LC-OLF-D6LC00061D-s001 [file LC-OLF-D6LC00061D-s001.pdf]

Supplementary Information (SI) for Lab on a Chip.
This journal is © The Royal Society of Chemistry 2026

Centrifugal microfluidics for rapid target analyte quantification in airborne bioaerosol

Soongwon Cho[a,b,c,1], Michael Brothers[d,e,1], Ziyu Chen[f,g,1], Samet Şahin[h,1], Yirui Xiong[a,f], Nicole Schaeublin[d,e], Douglas Adkins[i], Charles Call[j], Anthony Banks[a,k], Steve S. Kim[d,*], John A. Rogers[a,b,f,l,m*]

[a]Querrey Simpson Institute for Bioelectronics, Northwestern University, Evanston, IL, 60208, USA.
[b]Center for Bio-Integrated Electronics, Northwestern University, Evanston, IL, 60208, USA.
[c]Department of Chemical Engineering, College of Engineering, Kyung Hee University, Yongin, 17104, Republic of Korea
[d]Air Force Research Laboratory 711th Human Performance Wing, Wright Patterson Air Force Base, Dayton, OH, 45432, USA.
[e]BlueHalo, 4401 Dayton-Xenia Road, Dayton, OH, 45432, USA.
[f]Department of Material Science and Engineering, Northwestern University, Evanston, IL, 60208, USA.
[g]College of Engineering and Computer Science, The Australian National University, Canberra, Australian Capital.
[h]School of Engineering, Lancaster University, Lancaster, LA1 4YW, UK.
[i]Defiant Technologies, 6814 Academy Pkwy W NE, Albuquerque, NM, 87109, USA.
[j]Bioflyte, 10510 Research Rd SE #100, Albuquerque, NM, 87123, USA.
[k]Wearifi, 191 Waukegan RD Ste 365, Northfield, IL, 60093, USA.
[l]Department of Biomedical Engineering, Northwestern University, Evanston, IL, 60208, USA.
[m]Department of Neurological Surgery, Northwestern University; Evanston, IL, 60208, USA.

*Correspondence and requests for materials should be addressed to S.S. Kim (email: steve.kim.13@us.af.mil) and J.A.R. (e-mail: and jrogers@northwestern.edu)

[1]These authors contributed equally to this work.

Simplified beads-based ELISA in Eppendorf tube

The simplified beads-based ELISA in Eppendorf tubes was used to validate the bead-based assay chemistry that forms the basis of the centrifugal microfluidic ELISA. The assay generated a clear standard calibration curve using capture antibody conjugated PS beads (**Fig. S1a**). The tube-based assay process is analogous to the centrifugal microfluidic assay. PS beads with conjugated capture antibodies are first loaded into the tube, followed by coincubation with HSA, biotinylated detection antibody, and streptavidin-HRP. After washing, bead-bound streptavidin-HRP catalyzes TMB/H_2O_2 oxidation which is quantified by electrochemical chronoamperometric detection (**Fig. S1a**).

To assess particle associated assay interference relevant to field deployment, AuNPs were used as model particulate interferents to evaluate their effect on HRP-mediated TMB electrochemical detection, and non-specific adsorption to PS beads. For the electrochemical interference test, streptavidin-HRP, TMB/H_2O_2, and AuNPs were mixed in a one-pot reaction and incubated for 15 minutes. The resulting signal was measured by chronoamperometry (**Fig. S1c**). The comparable charge values across different AuNP volume percentages indicate that AuNPs do not substantially interfere with HRP-mediated TMB redox reaction or electrochemical readout under the tested conditions.

To evaluate non-specific adsorption, tube-based bead ELISA experiments were performed using 0 ng/ml HSA in the presence of AuNPs with and without BSA blocking. AuNPs without BSA blocking produced an increased background signal, consistent with non-specific interactions among AuNPs, HRP-containing assay components, and PS beads (**Fig. S1d**). BSA blocking reduced this non-specific background to a level comparable to the no-AuNP control. Optical images of PS beads further supported this result. PS beads exposed to unblocked AuNPs showed a visible color change, indicating non-specific adsorption of AuNPs onto the bead surface (**Fig. S1e**). In contrast, beads exposed to BSA-blocked AuNPs showed minimal color change, suggesting reduced non-specific adsorption.

Trial	Single stage impactor	Sample rinse		Wash rinse
1	Aerosol release	30 sec	Captured sample in 170 µL	Sampler vial wash 170-190 µL
	Sampling time	60 sec		
	Aerosol released weight	~ 50 mg		
	Captured protein	A_{562} = 0.250<br>7.16 µg		A_{562} = 0.080<br>1.72 µg
2	Aerosol release	30 sec	Captured sample in 190 µL	Sampler vial wash 200 µL
	Sampling time	60 sec		
	Aerosol released weight	~ 50 mg		
	Captured protein	A_{562} = 0.310<br>8.24 µg		A_{562} = 0.109<br>2.85 µg

Table S1. Concentration of captured HSA with a single-stage collector and an automated rinse system. A total of 75 mg of HSA was released into the 0.2 m3 test chamber.

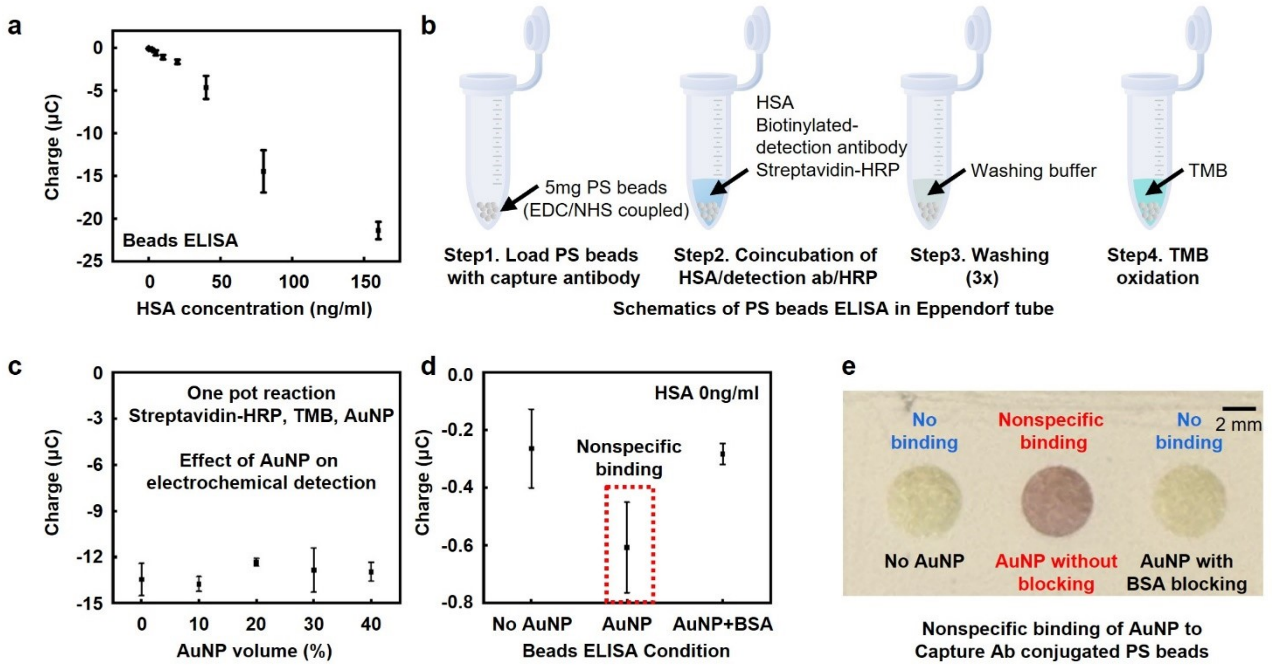

Fig. S1. Schematic illustrations and characterization of ELISA conducted in an Eppendorf tube using PS beads functionalized via EDC-NHS coupling chemistry. a) Standard calibration curve for PS beads-based ELISA. *n*=3. b) Schematic illustration of the carboxylated PS beads-based ELISA process for validation of EDC-NHS coupling conjugation chemistry and PS beads functionality for sensing HSA. c) Effect of gold nanoparticle concentrations on HRP-mediated TMB redox chemistry and chronoamperometric detection. d) Effect of AuNP blocking on non-specific background in a beads-based ELISA format. e) Optical image of PS beads after exposure to AuNPs with and without BSA blocking. Scale bar, 2 mm.

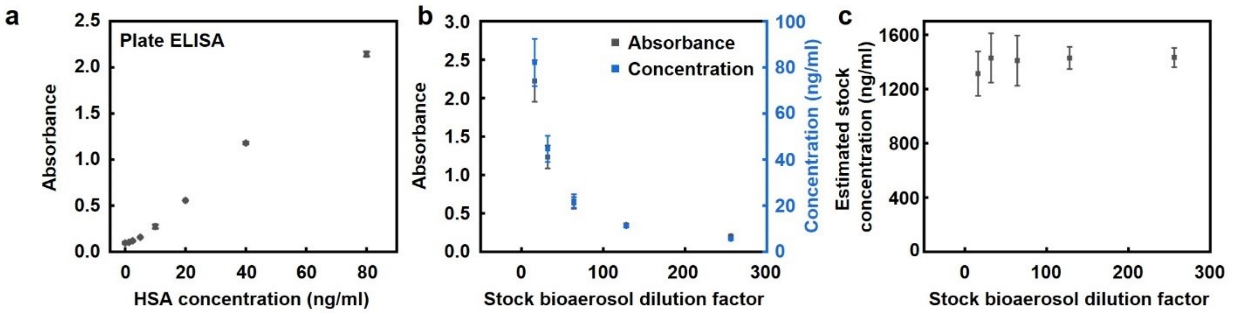

Fig. S2. Quantitative estimation of stock bioaerosol concentrations using plate ELISA. a) Standard calibration curve for plate ELISA (gold standard) for quantification of HSA. *n*=3. b) Absorbance (black) and HSA concentration (blue) versus the dilution factor of stock bioaerosol for determination of optimal dilution factor for quantification of stock bioaerosol HSA concentration. *n*=3. c) Estimation of HSA concentrations within a stock bioaerosol sample at different dilution factors by plate ELISA demonstrating reliable estimation. *n*=3.

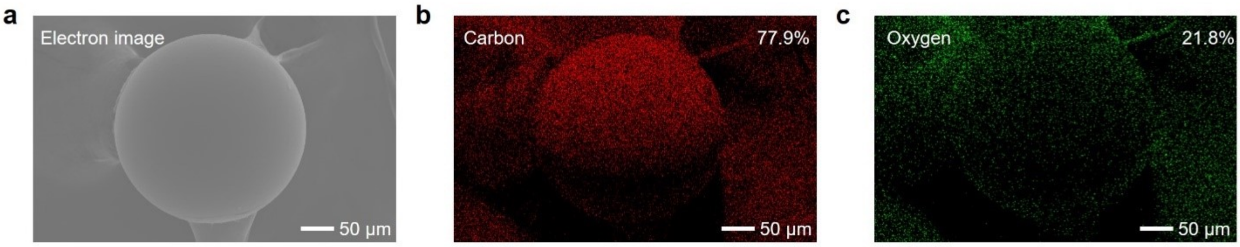

Fig. S3. EDS-SEM characterization of carboxylated PS beads. a) Scanning electron microscope (SEM) image of a carboxylated PS bead. Scale bar, 50 µm. b) Carbon energy dispersive x-ray spectroscopy (EDS) mapping for the carboxylated PS bead. Scale bar, 50 µm. c) Oxygen EDS mapping for the carboxylated PS bead. Scale bar, 50 µm.

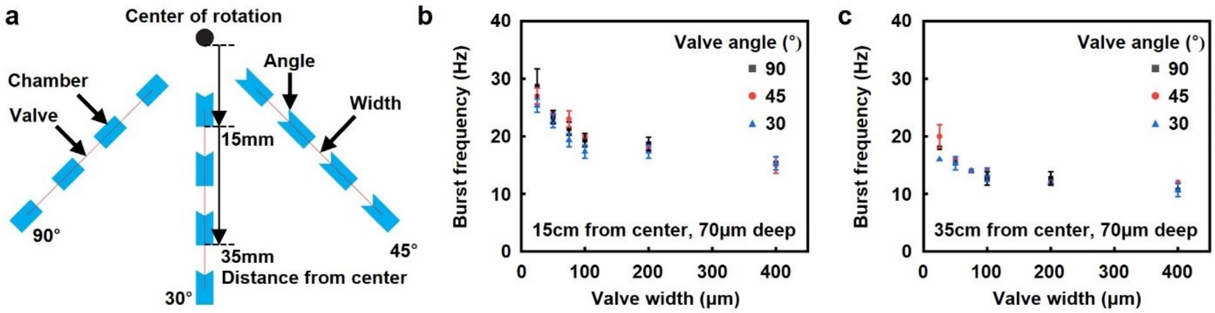

Fig. S4. Characterization of capillary valves burst frequency with respect to design, location, and width. a) Schematic illustration of the capillary valve designs tested for calibration of burst frequencies for designing the fully integrated device. b) Calibration of burst frequencies of capillary valves at different valve widths and angles at 15 cm from the center of rotation. *n*=3. c) Calibration of burst frequencies of capillary valves at different valve widths and angles at 35 cm from the center of rotation. *n*=3.

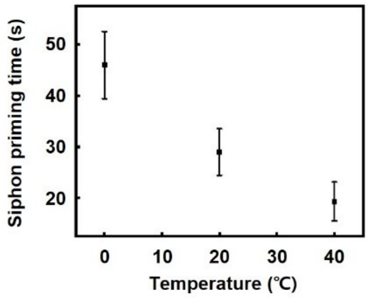

Fig. S5. Effect of temperature on siphon priming time. Siphon priming time was measured at different temperatures for valves coated with 0.02% PVA using 0.75 μL of liquid per valve.
